# Modelling modifiable factors associated with the probability of human rabies deaths among self-reported victims of dog bites in Abuja, Nigeria

**DOI:** 10.1371/journal.pntd.0011147

**Published:** 2023-02-21

**Authors:** Philip P. Mshelbwala, Ricardo J. Soares Magalhães, J. Scott Weese, Nasir O. Ahmed, Charles E. Rupprecht, Nicholas J. Clark

**Affiliations:** 1 School of Veterinary Science, The University of Queensland, Gatton, Australia; 2 Department of Veterinary Medicine, Faculty of Veterinary Medicine, University of Abuja, Abuja, Nigeria; 3 Children’s Health and Environment Program, UQ Children’s Health Research Centre, The University of Queensland, South Brisbane, Australia; 4 Department of Pathobiology, Ontario Veterinary College, Guelph, Canada; 5 Nigeria Centre for Disease Control, Abuja, Nigeria; 6 LYSSA LLC, Atlanta, Georgia, United States of America; 7 Auburn University, Auburn, Alabama, United States of America; Swiss Tropical and Public Health Institute: Schweizerisches Tropen- und Public Health-Institut, SWITZERLAND

## Abstract

Canine-mediated rabies kills tens of thousands of people annually in lesser-developed communities of Asia, Africa, and the Americas, primarily through bites from infected dogs. Multiple rabies outbreaks have been associated with human deaths in Nigeria. However, the lack of quality data on human rabies hinders advocacy and resource allocation for effective prevention and control. We obtained 20 years of dog bite surveillance data across 19 major hospitals in Abuja, incorporating modifiable and environmental covariates. To overcome the challenge of missing information, we used a Bayesian approach with expert-solicited prior information to jointly model missing covariate data and the additive effects of the covariates on the predicted probability of human death after rabies virus exposure. Only 1155 cases of dog bites were recorded throughout the study period, out of which 4.2% (N = 49) died of rabies. The odds for risk of human death were predicted to decrease among individuals who were bitten by owned dogs compared to those bitten by free-roaming dogs. Similarly, there was a predicted decrease in the probability of human death among victims bitten by vaccinated dogs compared to those bitten by unvaccinated dogs. The odds for the risk of human death after bitten individuals received rabies prophylaxis were predicted to decrease compared to no prophylaxis. We demonstrate the practical application of a regularised Bayesian approach to model sparse dog bite surveillance data to uncover risk factors for human rabies, with broader applications in other endemic rabies settings with similar profiles. The low reporting observed in this study underscores the need for community engagement and investment in surveillance to increase data availability. Better data on bite cases will help to estimate the burden of rabies in Nigeria and would be important to plan effective prevention and control of this disease.

## Author summary

Rabies results in tens of thousands of human deaths and tens of millions of exposures every year. Human rabies mediated by dogs is targeted for elimination by 2030. However, poor quality data hinder advocacy and resource allocation for effective prevention and control. We used a novel Bayesian approach incorporating domain expertise and realistic uncertainty to guide multiple imputations for missing data and understand factors associated with human rabies deaths among self-reported victims of dog bite in Abuja, Nigeria. Over 20 years, we observed low reporting across 19 major hospitals in the Federal Capital Territory, Abuja. The probability of rabies deaths after a dog bite was predicted to decrease after bites by owned dogs compared to free-roaming dogs. Similarly, there was a predicted decrease in the probability of human rabies death among victims bitten by vaccinated dogs compared to unvaccinated dogs. The odds for the risk of human rabies death after bitten individuals received human rabies vaccination were predicted to decrease compared to those without vaccination. Our analysis has shown how to approach missing rabies data objectively and transparently, which is valuable for gaining inference using real-world data. Our modelling approach will be helpful for future applied research in Nigeria and low and medium-income countries with similar challenges. The low reporting noted in this study underscores the need to improve rabies surveillance in Nigeria to understand the disease burden for effective prevention and control.

## 1 Introduction

Canine-mediated rabies is a model zoonosis for transdisciplinary One Health research into infections caused by rabies virus (RABV) and other lyssaviruses [1]. More than 20,000 people are annually estimated to die from rabies in Africa, with over 95% mortalities attributed to dog bites [2,3]. Children, the underprivileged, and those residing in rural areas are disproportionately affected because of unsafe dog ownership practices, poor access to healthcare, and low levels of health education [4,5]. Human rabies can be prevented with appropriate knowledge of transmission pathways, avoidance of viral exposure, or access to appropriate rabies postexposure prophylaxis (PEP) after a dog bite, including a thorough understanding of modifiable factors driving RABV transmission. Nevertheless, rabies remains neglected due to a lack of quality data to quantify the disease burden to support targeted prevention and control efforts toward the global ‘Zero by Thirty’ (ZBT) goal [6].

Due to its invariably fatal outcome, achieving canine-mediated rabies elimination requires a sustained reduction in human fatalities through a One Health approach to epidemiologic analyses and understanding of factors driving RABV transmission [1]. Previous studies have used data on dog bite occurrences to estimate the number of human rabies deaths [3,7–9]. Such data may be utilised to identify locally relevant modifiable factors associated with RABV perpetuation to break the transmission cycle and avert such deaths.

Several studies have described the occurrence of dog bites in human and veterinary settings in Nigeria [5]. Towards the current ZBT target, effective rabies prevention requires information on how the risk of rabies deaths is associated with local modifiable factors. However, no study in Nigeria have modelled the risk of dog bite-associated deaths. For context, across most Nigerian healthcare facilities, PEP is not readily available or free [10]. Some dog bite victims die from rabies due to the inability to pay for PEP, as well as the distance to a hospital to access such health care [11].

Abuja is the capital of Nigeria, located in the centre of the country within the Federal Capital Territory (FCT), with six area councils, namely Abaji, Bwari, Gwagwalada, Kuje, Kwali, and Abuja Municipal (AMAC). As of the last census in 2006, the FCT was Nigeria’s 18th most populous city. According to the United Nations, the FCT was the fastest growing city in the world between 2000 and 2010, with a 139.7% growth rate(https://worldpopulationreview.com/world-cities/abuja-population).

The FCT experiences multiple rabies outbreaks, with associated human deaths [12]. Previous studies in the FCT indicated an increase in dog bites, a low (i.e., 38%) dog vaccination rate across semi-urban areas compared to the recommended 70%, an abundance of free-ranging dogs, and the presence of RABV in dogs slaughtered for human consumption [13–15]. Data on the size of the dog population for the whole FCT is unavailable, but a study conducted in Gwagwalada estimated 103,758 dogs [15]. Given the high RABV transmission risk in the FCT, there is a need to understand factors associated with the probability of human deaths, using available evidence from clinics and hospitals. The FCT has several functional health facilities, where record-keeping for dog bite cases is better managed, compared to other states. Therefore, the data quality is assumed to be at a higher standard. However, in the context of national ZBT efforts, poor-quality data have been shown to hinder advocacy and resource allocation for effective rabies prevention and control [1,5,16].

A recent study showed that even with a large proportion of missing data (up to 90%), multiple imputations could provide an unbiased estimate with improved efficiency, so long as the model is specified correctly [17]. We report how we overcame the challenge of missing information in Nigeria using a probabilistic statistical model that incorporates domain expertise and realistic uncertainty to model the additive effects of modifiable covariates on the predictive probability of human rabies deaths. Our population of interest was individuals who sought health care following a dog bite. Our objective was to develop a model-based approach to understand the risk of human rabies deaths in this specific cohort using data from 19 major health facilities in the FCT.

## 2 Materials and methods

### 2.1 Ethics approval for the study

The Federal Capital Territory Health Research Ethics Committee (FCT HREC) approved the study and was ratified by the University of Queensland Research Ethics Committee 2019001486 /FHREC/2019/01/04/21-01-19. No victim identifiers were present in this paper.

### 2.2 Data on victims of dog bites in Abuja

We performed a retrospective study of self-reported dog bite occurrence in all 14 secondary healthcare facilities in the FCT, one tertiary facility (Gwagwalada), three private facilities (Gwagwalada, AMAC and Bwari), and one Primary facility (AMAC) between January 2000 and December 2018.

To begin the study, Disease Surveillance and Notification Officers attached to the facility and student volunteers, reviewed all dog bite cases reported to the accident and emergency unit from the registers. Secondly, they retrieved all files and extracted the following information on the dog bite victim: age, gender, occupation, victims’ address, site of the bite, severity of the bite, reported use of PEP (number of vaccination doses taken by the victim). The PEP data were further classified into two categories for analysis (i.e., those who took at least one vaccine dose and those who failed to take any doses), the ownership and vaccination status of the offending dog, and the fate of the victim (whether or not the victim died showing clinical signs consistent with rabies as documented by the attending clinician (i.e. probable based on WHO classification) [18]. For example, all 49 victims who died had documented clinical notes. Of these, 9 received PEP while showing clinical signs of rabies. In addition, we attempted to confirm the fate of other victims using phone calls. However, due to logistic challenges and initial apprehension, we relied primarily on the clinical notes. Any victim who returned to the clinic for other health conditions, or responded to our phone calls, was deemed alive. We discarded information for victims who had no follow-up information and those for whom we could not ascertain the status of the victims via phone calls. Victims who died showing clinical signs consistent with rabies were classified as zero, while those alive were classified as one. All bite victims who showed clinical signs while taking PEP were considered unvaccinated. Confirmatory laboratory diagnosis for human rabies is unlikely across West Africa and Nigeria [5,19]. Hence, we based our outcome of interest on the victim’s binary fate.

For the offending dogs, only six had a confirmatory rabies diagnosis. For the ownership status of dogs, we relied on the history provided by the victims as documented by the physician. We classified dogs as free-roaming if the clinical notes indicated they were free-roaming or stray. It is likely that those dogs were owned who were allowed to roam freely, because a previous report indicated only a 3% minority of dogs are feral in Africa [20]. All information was entered manually into a standardised data abstraction tool. Finally, data were transferred into SPSS, exported to Microsoft Excel, and cleaned for analysis. We attempted to obtain data from the veterinary hospitals in the FCT and the Federal Ministry of Agriculture to supplement the data, because a study reported that Nigerian bite victims often report to the veterinary hospital [21]. However, no records were found from the clinics and the Federal Ministry of Agriculture, in agreement with a report of a study in Ogun State, Nigeria, indicated that record keeping in a veterinary hospital was poor [21]. We also had no information to evaluate the risk of rabies in the offending dog.

### 2.3 Environmental, socioeconomic, and epidemiological variables

Human rabies is associated with sociodemographic and ecological factors that bring people into close contact with infected dogs [22]. Previous studies showed that poverty, population density, literacy, distance to the road network, and residing in a rural area were associated with canine and human rabies [22,23]. For this study, we obtained several maps: a poverty raster map (approximately 1km resolution) of the proportion of people per grid square living in poverty in the FCT [20]; a literacy raster map (approximately 1km resolution) showing the proportion of men and women aged 15–49 per grid square as literate in the FCT [21]; an urban extent grid (v.1) showing the proportion of rural and urban areas in the LGA, extracted from the Global Rural-Urban Mapping Project (GRUMP v.1) [24]; and a population density map at 30 arc-second (~1 km at the equator) resolution from the World Pop project [25]. We used a 20-year average for land cover and population density. In contrast, for literacy and poverty, we used data from 2013, and the current distance to the veterinary clinic and hospital. We assumed no significant changes in the number of Government human and veterinary hospitals where dog victims were likely to report in the event of a dog bite, because the cost of care is relatively affordable in a government compared to a private hospital [26].

Previous studies in Nigeria showed that 27% of dog bite victims reported to a veterinary clinic, while the rest reported to a human hospital [5]. The distance to a veterinary clinic or hospital was used as proxy for PEP access. To derive a map of distances to the veterinary clinic, we obtained the coordinates of all H.F. in the FCT from the Federal Ministry of Health [27] and geolocated veterinary hospitals/clinics in Nigeria using Google Earth (https://earth.google.com/web/). We then calculated the Euclidean distance between human dog bite cases to a hospital/clinic and veterinary clinics in ArcGIS version 10.8. We also obtained a road network raster from DIVA-GIS (https://www.diva-gis.org free/gdata) and calculated the mean distance to the road network in meters. We scaled all continuous variables to unit variance by subtracting the mean and dividing by one standard deviation.

## 3 Statistical analyses

### 3.1 Model definition

Bayesian inference allows the estimation of a joint posterior distribution over unknown parameters for a statistical model, which is the product of the prior distribution and the likelihood of the data. Our analysis was conducted with R version 4.1.3, using the rjags package, which provides an interphase for JAGS (Just Another Gibbs Sampler) library to perform Bayesian analysis [28]. Our model was constructed considering that the observed vector of human deaths after a dog bite in Abuja was drawn from a Bernoulli distribution with unknown parameters *p*, the probability of human rabies death. We modelled *p* using a logit link function and linear predictor to estimate the additive effects of covariates on the probability of a human rabies death. The number of parameters to be estimated was 18, including the intercept and a vector of individual, sociodemographic, and landscape predictors:

logit(p(Y = 1)) = β0 + β1*Gender+ β2*Age + β3*Site of bite + β4*Bite type+ β5*Category of bite + β6*PEP + β7*Dog ownership+ β8*Dog vaccination status + β9*Distance to a hospital + β10*Distance to a veterinary hospital + β11*Urban/rural location + β12*Population density + β13*Poverty level + β14*Literacy level + β15*Distance to road + β16*Grass + β17*Shrubland, where Y is a binary indicator of human rabies deaths following dog bite (1 if death and 0 alive).

### 3.2 Accounting for missing data in the predictors of rabies deaths

For covariates with missing data, including the site of the bite (44%, 506/1,155) and vaccination status of the offending dogs (68.3%, 789/1,155), we used prior information/evidence from the literature on dog vaccination rates in the FCT and general rabies epidemiology in Nigeria to model missing values [5,29]. For dogs whose victims could not identify an owner, we termed an unknown dog with missing information on vaccination status. We drew binary values from a Bernoulli distribution, assuming that each dog had a 38% chance of being vaccinated, using evidence from a recent Abuja study that reported vaccination coverage for owned dogs [15]. We drew missing values for the site of bite from a categorical distribution, setting relative prior weights of 2-1-2 for a bite to the lower limb, head or multiple sites, because historical data and previous reports from Nigeria indicate victims are more likely to be bitten on the lower limb and multiple locations than the head [29]. An interaction term between the site of the bite and the victim’s age was initially included (assuming the site of the bite depended on the victim’s age). However, chains mixed poorly, and Markov Chain Monte Carlo (MCMC) diagnostics suggested the data were not informative to accurately estimate marginal and interaction effects. Prior to modelling, we carried out a pairwise correlation to measure our predictor variables using the cor() command in R and removed highly collinear variables in our model.

### 3.3 Accounting for sparse rabies death outcome data and multiple predictors

Due to the large number of candidate covariates and our prior assumption that most effects on a patient’s probability of death due to rabies will be near-zero and challenging to estimate with finite outcome rabies death data, we guarded against overfitting by trialling two regularisation priors for the β parameters. Regularisation aims to shrink uninformative coefficients towards zero to produce a sparser and more parsimonious model, while accounting for interdependencies between the regularised set of predictors. First, we fit a model that used a modified version of the regularised horseshoe prior [30] for the βs, which was defined as follows:

βj ∼ Normal(Mean = 0, SD = λ * τ2j)

λ ∼ half-Cauchy(Mean = 0, DF = 1)

τ2j ∼ Gamma(Shape = 0.1, Rate = 0.001),

A regularised horseshoe prior tends to shrink small coefficients towards zero by combination of a global shrinkage penalty (λ) and coefficient-specific penalties (τ2j). The prior density is symmetric about zero but incorporates fat tails and a large spike at zero, making it suitable for models with many regression coefficients where only a minority of these coefficients is expected to be non-zero. However, as the regularised horseshoe can strongly pull effects toward zero, it can be challenging to estimate effects with probability mass at small, non-zero values accurately. Therefore, we trialled a less informative regularising prior for the βs by placing a double exponential (i.e., Laplace) prior on these parameters. A Laplace prior is loosely regarded as the Bayesian equivalent of L1 regularisation (LASSO penalty), simultaneously allowing for many zero effects and some large non-zero effects and is defined by: y ~ Laplace (Mean = 0, Precision = 1).

### 3.4 Parameter estimation

We used a MCMC Gibbs sampling algorithm to sample from the joint posterior of unknown model parameters for each of the above prior specifications. Estimation was conducted in the software JAGS using the R interface rjags [28]. A total of four MCMC chains were run for 5,000 iterations for burn-in, discarded—visual inspection of the posterior density and trace plots was used to note convergence after 100,000 post burn-in iterations. Following convergence, we stored the posterior distributions from model parameters for inference and calculated the summaries of the parameters, including posterior means and 95% credible intervals (Crl).

Posterior predictive checking is designed to monitor the quality of a statistical model by detecting any inherent difference between the model-simulated data and the observed data through the generation of replicated data sets from the posterior distribution and comparing the observed data for a feature of interest [9]. To conduct predictive checks for each of the above models, we simulated Bernoulli draws from the model’s estimated probability of rabies death for each individual and compared these simulations to the observed data to check for systematic inconsistencies between the observations and model assumptions. A Bayesian p-value was calculated as the proportion of MCMC iterations in which the sum of the simulated Bernoulli vector (the model’s estimated total number of deaths) was larger than the sum of the corresponding observed vector (the true total number of deaths). A model with a Bayesian p-value close to 0.5 indicates a lack of systematic discrepancy between the observed data and the model’s assumed data generating process. We also compared model fits using the penalised deviance information criterion, which is estimated by adding the effective number of parameters (Pd) to the expected deviance [31]. Superior model fitting was evidenced by the lower deviance information criterion (DIC).

### 3.5 Posterior visualisation of key parameters

We used marginal posterior simulations to understand how the probability of human death due to rabies was expected to change across different magnitudes/levels for modifiable covariates. We simulated how much the probability of death was expected to change when a dog bite victim took at least one dose of vaccine (or none) and when the dog bite victim took varying vaccine doses (and none). We simulated how much the probability of death was expected to change when the offending dogs were vaccinated vs. unvaccinated and when owned vs. free roaming.

### 3.6 Residual spatial autocorrelation

To gain insight into any evidence for spatial dependence left unexplained by the model, we calculated randomised quantile (Dunn-Smyth) residuals for each posterior simulation and inspected these residuals for possible spatial autocorrelation. Briefly, we computed randomised quantile residuals, which samples variability in the parameter estimates by inverting the fitted distribution function for each response value and finding the equivalent standard normal [9]. We fit semi-variograms to each posterior draw of these residuals. A semi-variogram is described by nugget effect, range, and partial sill [32]. Nugget represents a small-scale data variability. The range is the distance the data are no longer correlated. The partial sill is the semivariance due to spatial dependence. The nugget plus the partial sill is also known as the total variance or sill [32]. We calculated the nugget:sill ratio for each posterior semi-variogram, revealing the overall variance at a distance smaller than the smallest lag interval and how much variance was accounted for in the model. A nugget:sill ratio of <0.25 indicates strong residual spatial dependence, 0.25–0.75 shows moderate spatial dependence, and >0.75 indicates weak spatial dependency [33].

### 3.7 Sensitivity analysis

Sensitivity analysis is a way of checking the final model result based on the reference prior (original) concerning the results obtained using different priors to aid in interpreting results transparently [34]. To examine the effect of our prior imputations for dog vaccination with large numbers of missing values, we conducted a sensitivity analysis by building four models that made different assumptions about vaccination probability for missing values. In the first model, we adjusted the probability of dog vaccination to 35% to account for 3% of the free-roaming dog population out of the initial 38% because a previous report indicated that only 3% of African dogs are strays [18]. In the second model, we allowed the probability of vaccination to vary (i.e., between 20% and 45%) in each MCMC iteration. In the third model, we incorporated slightly more realism by allowing owned and free-roaming dogs to have different vaccination probabilities, with values between 35% and 60% for owned dogs and 30% for free-roaming dogs. For the last model, we varied the probabilities for both owned and free-roaming dogs, with values between 25% and 75% for owned dogs and between 20%- and 45% for free-roaming dogs. We compared posterior contrast distributions from each model to see how these prior choices impacted our resulting inferences.

## 4 Results

### 4.1 Demographic and health characteristics of dog bite victims

A total of 1,155 dog bite victims were reported in 19 H.F. across the FCT during 2000–2018, of which 4.2% (49/1,155) were reported to have died of rabies. Most victims reported to a secondary H.F. (93.2%, 1,076/1,155). Victims were predominantly male (66%, 756/1,155), between the age of 0–15 (40.3%, 465/1,155), and primarily students (44%, 519/1,155). Only a minority of biting dogs were vaccinated against rabies (14%, 166/1,155). The majority were free-roaming dogs, and most dog bite incidents were due to provoked bites by a person (85%, 980/1,155). Table 1 compares individual-level information distribution across victims who died or were alive after a dog bite.

**Table 1:** Distribution of variables among victims of dog bite who died or were alive following a dog bite in Abuja between 2000 and 2018.

Variables	Alive	Dead
**Gender**		
Male	727	29
Female	376	20
**Vaccine dose**		
Zero doses	212	49
One dose	96	0
Two doses	34	0
Three doses	87	0
Four doses	283	0
Five doses	396	0
**Age**		
0–15	448	17
16–30	280	13
31–45	309	18
>45	69	1
**Site of bite**		
Lower limb	300	5
Head and neck	68	5
Multiple sites	225	11
No data		
**Dog ownership**		
Owned	366	6
Free roaming/stray	789	46
**Dog vaccination**		
Vaccinated	166	2
Unvaccinated	200	14
No data	789	33
**Class of bite**		
Class 2	397	13
Class 3	709	38

### 4.2 Model selection and performance

Regarding the relative fits of the models to our observed data, most coefficients were drawn towards zero in model 1 (regularised horseshoe prior), though mixing of chains was poor for some parameters. This model also displayed a higher DIC (38086) and an inferior Bayesian p-value = 0.42. In contrast, model 2 (Laplace prior) showed good mixing of chains, reasonable Rhat split diagnostics, S1 File a lower DIC (38064), and a Bayesian p-value nearer to 0.5 (p = 0.46). We used model 2 for inference and subsequent analyses based on these results. Rhat refers to the potential scale reduction statistic, also known as the Gelman-Rubin statistic, which is the ratio of the variance of a parameter when the data are pooled across all the chains to the within-chain variance. Values of Rhat close to 1 indicate good performance of the sampler [35].

### 4.3 Factors associated with the probability of death of dog bite victims

**Fig 1:**
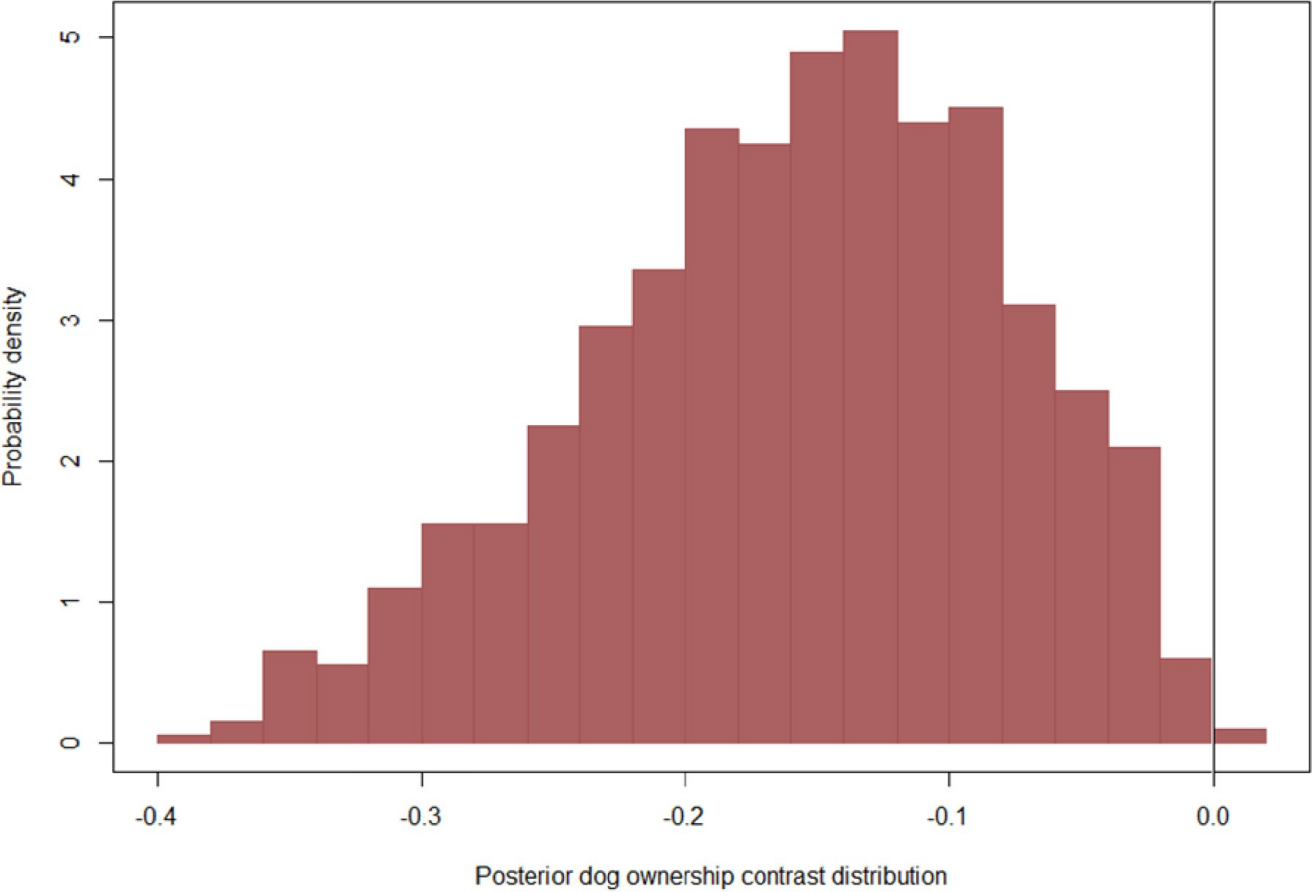
Posterior marginal contrast distribution demonstrating the estimated posterior probability of deaths was predicted to change when comparing bites from owned dogs to bites from free-roaming dogs.

**Fig 2:**
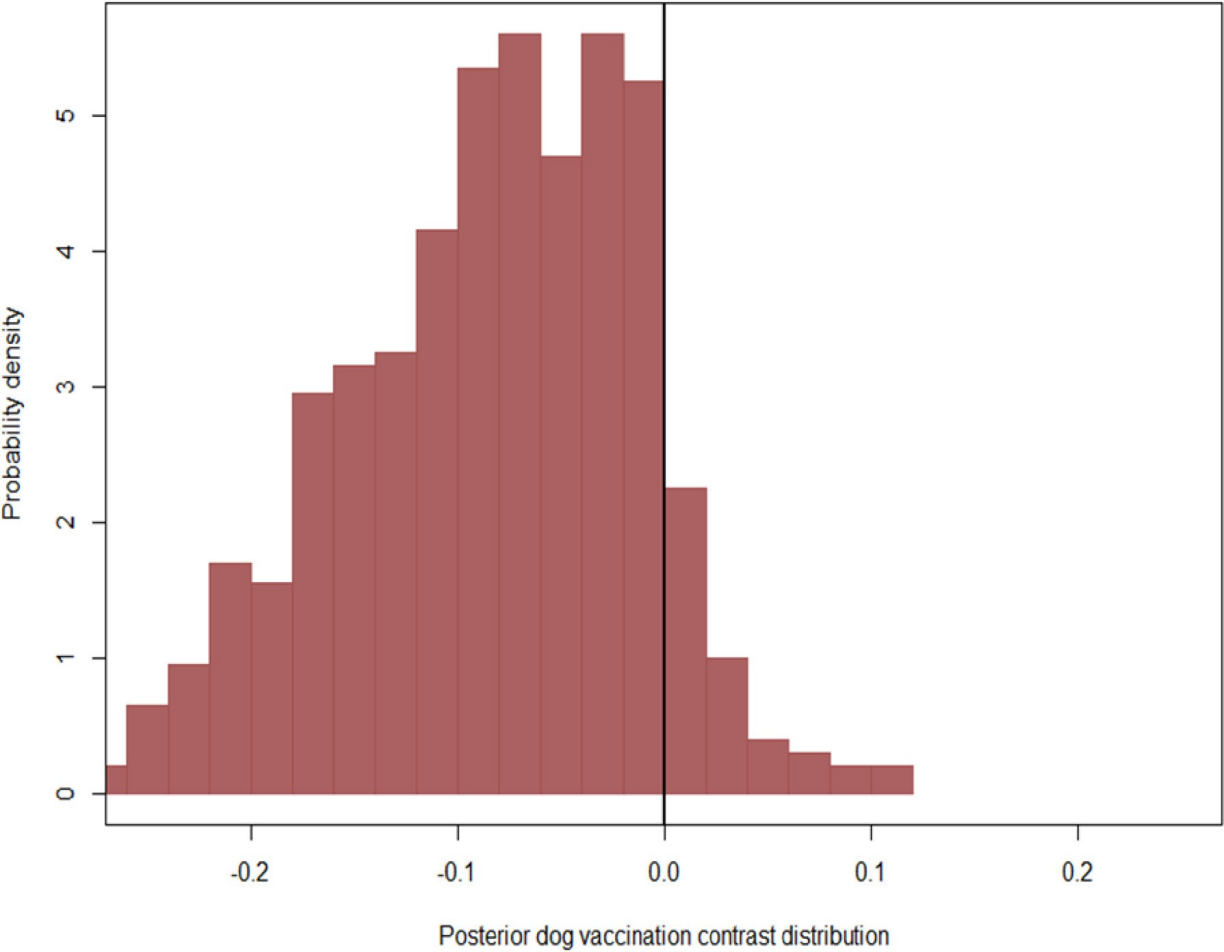
Posterior marginal contrast distribution demonstrating how the estimated posterior probability of deaths was predicted to change when comparing bites from unvaccinated dogs to bites from vaccinated dogs.

**Fig 3:**
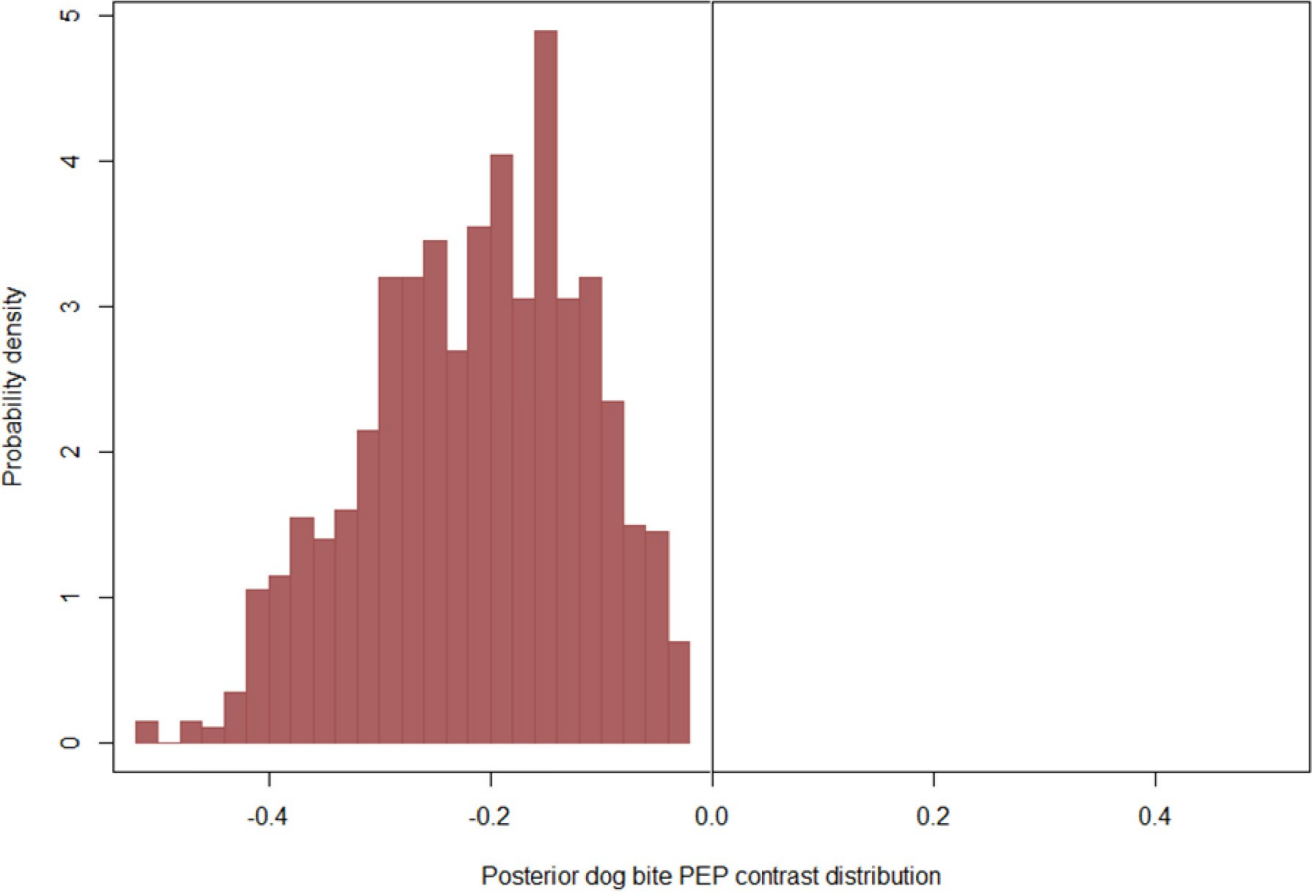
Posterior marginal contrast distribution demonstrating the estimated posterior probability of deaths was predicted to change when comparing victims who received PEP and those who did not.

Dog vaccination status, dog ownership, and victims who took rabies vaccination were estimated to have regression coefficients with a probability density predominately away from zero. Precisely, the probability of human rabies death was estimated to decline when comparing bites by owned vs. free-roaming dogs (Fig 1), for bites from vaccinated vs. unvaccinated dogs (Fig 2), and for victims who had PEP vs. no PEP (Fig 3). Other variables, including mean distance to the hospital, veterinary facility (proximity to a veterinary facility), population density, land covers, literacy level, poverty, count of rabies cases per ward, site of the bite and dog bite victim age, displayed weaker effects that had a substantial probability density near zero (S1 File). In contrast, the type of bite and urban locations were centred at zero (and considered non-significant herein, S1 File). Our results showed that individuals bitten by owned dogs were less likely to die of rabies (OR = 0.27, 95% CrI: 0.01–0.44) than those bitten by free-roaming dogs. Individuals bitten by vaccinated dogs were less likely to die from rabies (OR = 0.48, 95% CrI: 0.01–1.34) than those bitten by unvaccinated dogs. Individuals who received PEP were less likely (OR = 0.002, 95% CrI: 0.01–0.01) to die from rabies than those who had no PEP (Table 2).

**Table 2:** Posterior mean, standard deviation, credible intervals, and convergence diagnostics for estimated effects on the logit (probability of deaths) due to rabies after a dog bite.

Variable	Variable distribution	mean	sd	95% CrI		Rhat
Continuous	Mean distance to a hospital	0.404	0.335	-0.183	1.09	1
Continuous	Mean distance to a veterinary clinic	0.381	0.239	-0.072	0.859	1
Categorical	Bite type	-0.085	0.366	-0.837	0.607	1
Categorical	Age of victim	0.112	0.1956	-0.258	0.506	1
Categorical	Ownership status of dog	-1.794	0.522	-2.841	-0.813	1
Continuous	Population density	0.430	0.223	0.012	0.886	1
Continuous	Poverty	0.552	0.386	-0.129	1.348	1
Continuous	Grass	-0.586	0.362	-1.348	0.027	1
Continuous	Literacy level	-0.233	0.290	-0.848	0.279	1
Categorical	Urban locations	0.84	0.465	0.019	1.847	1
Categorical	Bite Type	0.037	0.469	-0.926	0.989	1
Continuous	Distance to road	0.183	0.23	-0.218	0.665	1
Continuous	Shrubland	0.675	0.326	0.054	1.333	1
Categorical	Vaccination status of dog	-2.167	0.751	-3.746	-0.805	1
Categorical	Gender of victim	-0.34	0.362	-1.085	0.306	1
Categorical	PEP	-6.232	1.055	-8.59	-4.55	1

Crl (Credible interval), sd (Standard deviation)

Posterior marginal contrast distributions highlight the non-normality of expected changes in the probability of human rabies deaths following potential interventions. In particular, our model estimated strongly left-skewed decreases in the probability of human rabies deaths when comparing bites from owned vs. free-roaming (Fig 1) and when comparing bites from unvaccinated vs. vaccinated dogs (Fig 2), suggesting decreases of up to 20% in probability were not considered likely, but also not implausible by the model. Our model also estimated a significant left-skewed decrease in the probability of human rabies deaths when comparing victims who received PEP and those who failed to receive PEP (Fig 3).

### 4.4 Residual spatial dependence

The posterior mean nugget:sill ratio was 0.824, suggesting there was weak evidence of remaining spatial dependency—that is, there was little spatial dependence in the data unaccounted for the variables in the model (i.e., that estimates of coefficient standard errors and effect sizes are unlikely biased by non-independence).

### 4.5 Sensitivity analysis

**Fig 4:**
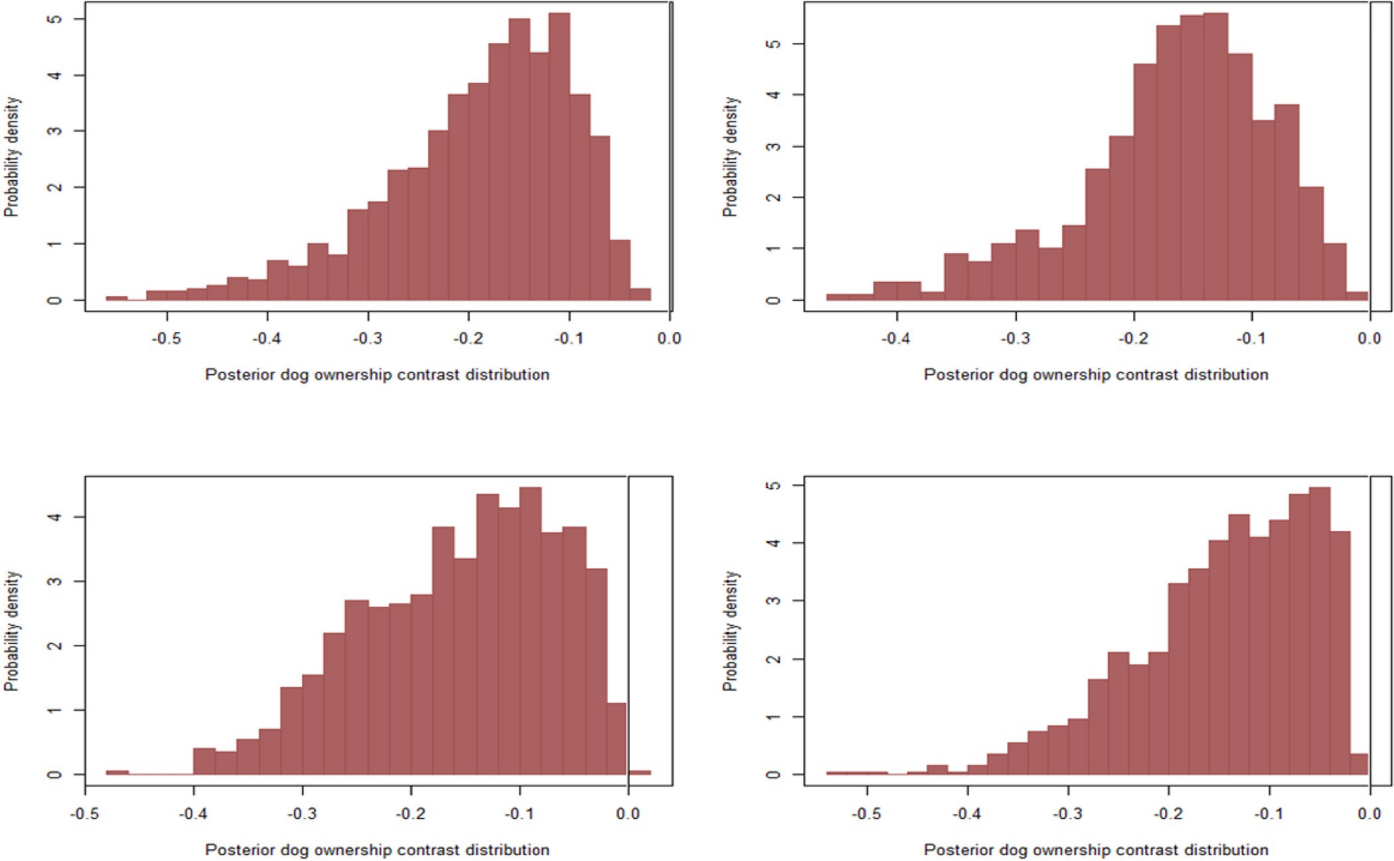
Posterior marginal contrast distribution demonstrating how the estimated probability of deaths was predicted to change, comparing bites from owned to free-roaming dogs at 35% vaccination probability to account for 3% of free-roaming (Top left), when the probability of vaccination was varied between 20% and 45% in each MCMC iteration(Top right), when the probability for owned dogs was varied between 35% and 60% and 30% for free-roaming dogs(Bottom left) and when we varied the probabilities between 25% - 75% for owned and 20%- 45% for free-roaming dogs(Bottom right).

After visual inspection of the posterior marginal plots, our results indicated non-normality of the expected changes in the probability of human rabies deaths following prior imputations at different vaccination and ownership probabilities with consistent results with the initial assumption. Our model estimated a strongly right-skewed decrease in the probability of human rabies deaths when comparing bites from owned vs free-roaming dogs at different vaccination probabilities between the ranges of 10% and 20% across all imputations, with a slightly more decrease when the probability for owned dogs was varied between 35% and 60% and 30% for free-roaming dogs (bottom left) and when we varied the probabilities between 25% - 75% for owned and 20%- 45% for free-roaming dogs (bottom right) (Fig 4).

**Fig 5:**
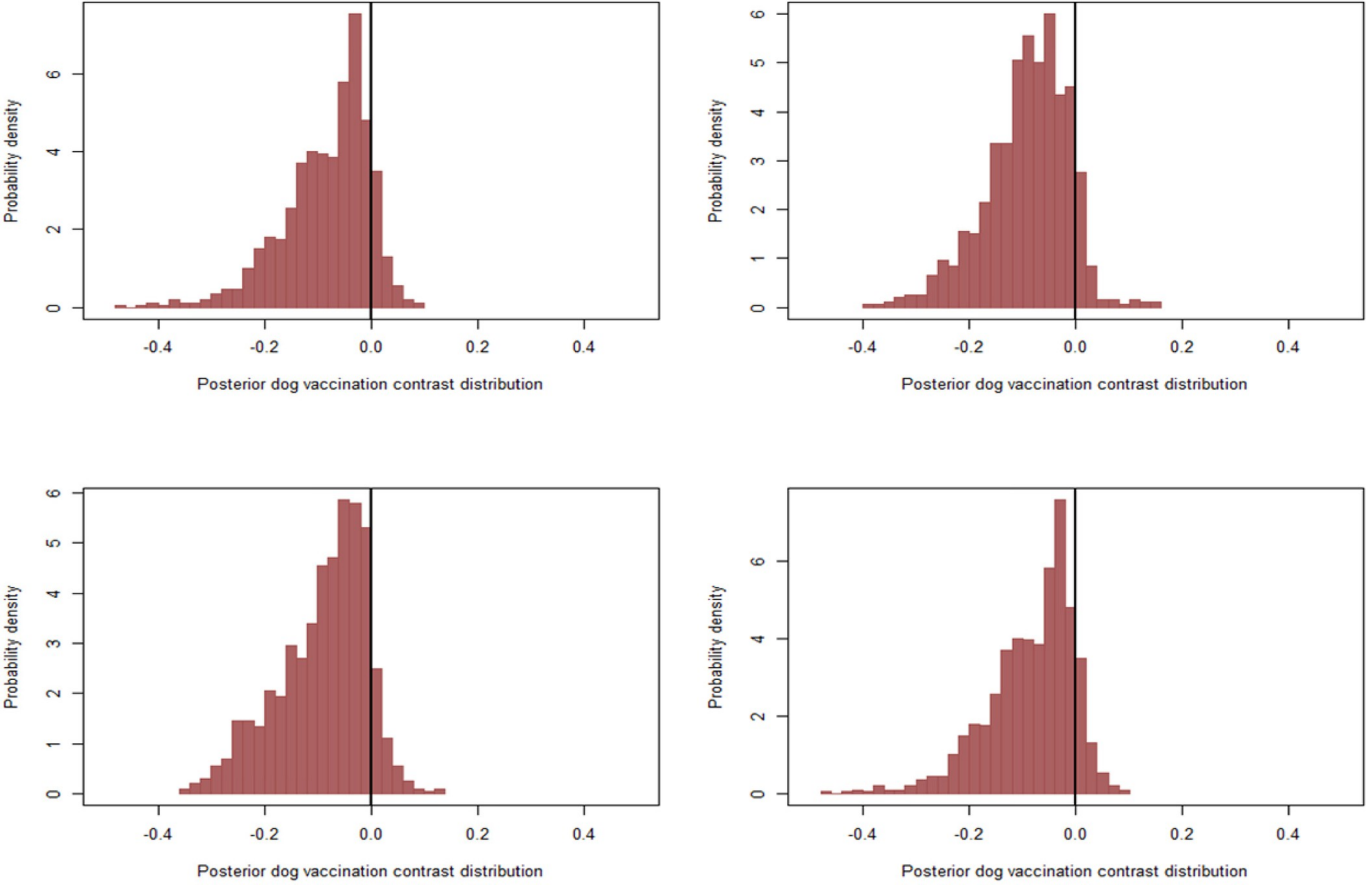
Posterior marginal contrast distribution demonstrating how the estimated probability of deaths was predicted to change when comparing bites from vaccinated to unvaccinated dogs, when the vaccination probability was adjusted to 35% to account for 3% of free-roaming dogs (Top left) when the probability of vaccination was varied between 20% and 45% in each MCMC iteration(Top right) when the probability for owned and free-roaming dogs were varied between 35% and 60% for owned dogs and 30% for free-roaming dogs(Button left) and we varied the probabilities between 25% - 75% for owned and 20%- 45% for free-roaming dogs(Button right).

**Fig 6:**
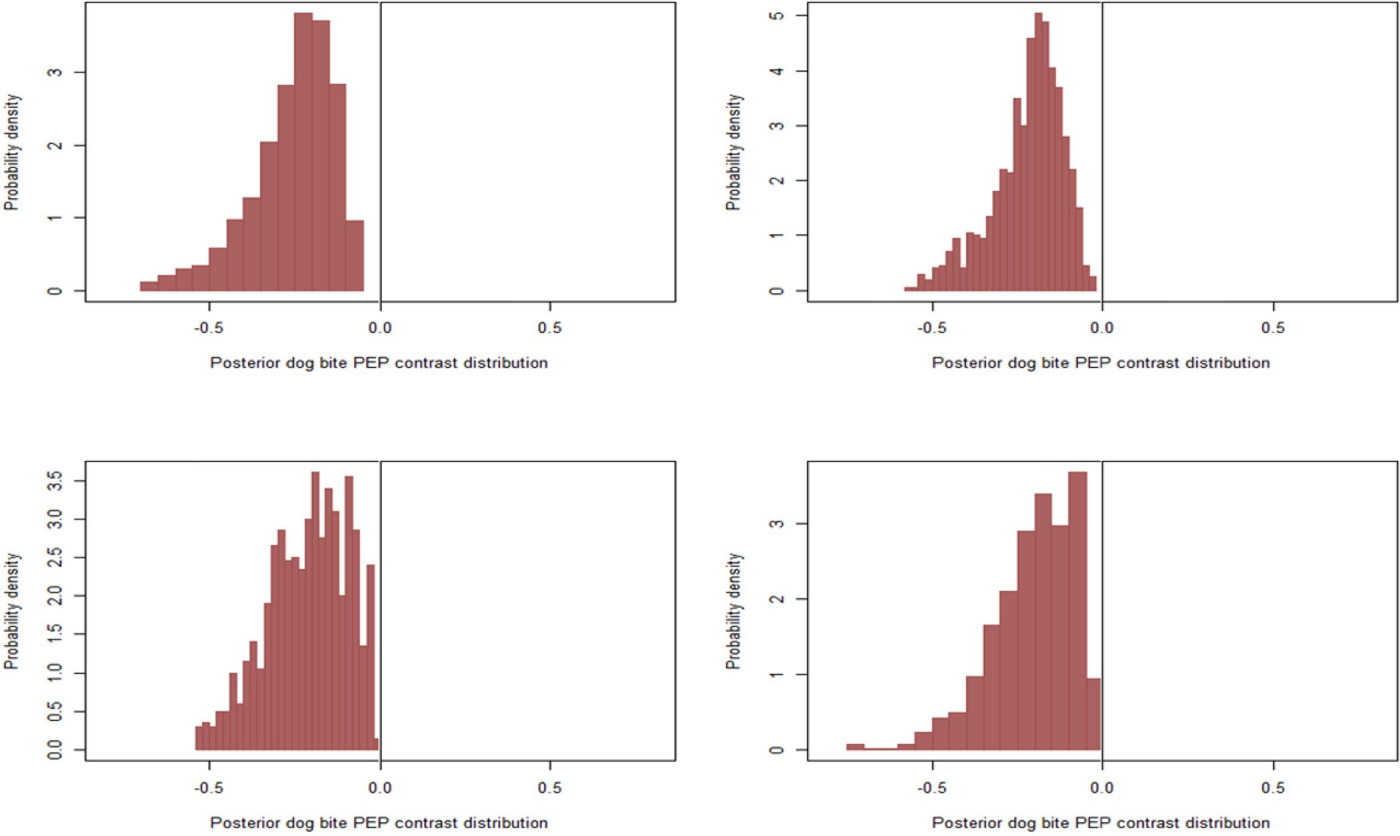
Posterior marginal contrast demonstrating how the estimated posterior probability of deaths was predicted to change when comparing victims who received PEP and those who did not at 35% vaccination probability to account for 3% of free-roaming dogs (Top left) when the probability of vaccination was varied between 20% and 45% in each MCMC iteration (Top right) when the probability for owned dogs was varied between 35% and 60% and 30% for free-roaming dogs (Button left) and when we varied the probabilities between 25% - 75% for owned and 20%- 45% for free-roaming dogs(button right).

When comparing bites from vaccinated vs unvaccinated dogs at different prior imputations, our models estimated a left-skewed decrease in the probability of human rabies across all prior imputations without much difference (Fig 5). Similarly, our models estimated a strong skewed decrease in the probability of human rabies deaths when comparing victims who received PEP vs those who did not, about 50% decrease, especially when the probability of vaccination was varied between 20% and 45% in each MCMC iteration (top right), when the probability for owned dogs was varied between 35% and 60% and 30% for free-roaming dogs (bottom left) and when we varied the probabilities between 25% - 75% for owned and 20%- 45% for free-roaming dogs (bottom right) (Fig 6). The consistency of our results across different prior configurations likely indicates that the observations with missing vaccination data were less informative for the likelihood overall.

## 5 Discussion

Our study used extremely sparse surveillance data to model factors associated with the probability of human rabies deaths after a dog bite in Nigeria, which is crucial, especially in risk assessments consistent with the ZBT goal. The lack of quality data on human rabies to quantify the rabies burden hinders advocacy and resource allocation for local prevention and control efforts. By applying an epidemiological modelling structure that accounts for data scarcity and interdependencies between rabies predictors, we identified modifiable risk factors associated with the probability of human rabies death after a dog bite in the FCT. While the findings of this study might not be generalisable to individuals that did not seek care in the FCT, we can glean some insight into why some rabies victims died. This is a valuable contribution to the field, especially for advocacy to local authorities in Nigeria (and elsewhere where rabies is endemic) to invest in rabies prevention and control. Owing to the predictable underreporting of rabies in such endemic settings, findings from this study can motivate future investigations into risk among individuals that did not report to a clinic to outline the best prevention and control strategies.

Our data indicated that at least 49 victims died, showing clinical signs consistent with rabies. While definitive diagnosis was lacking, the clinical signs and history of rabies exposure provide a reasonable presumptive diagnosis in a situation where definitive diagnosis is rarely possible. Rabies affects the poor in rural settings without access to health care, who often cannot afford PEP, are located distantly to a health facility, or are unaware of the need for PEP, and often utilise non-orthodox treatments [11]. Our detected cases are a distinct minority of the actual number of people who die due to rabies in Nigeria (and elsewhere with similar health disparities). Moreover, certain religious groups, such as Muslims, practice immediate burial after death and form a significant proportion of the FCT population. Human rabies victims are likely to be buried, thereby precluding diagnosis. As such, there is a need for more education on rabies and community engagement, including implementing an Integrated Bite Case Management strategy to prevent such deaths and allow a more thorough understanding of the actual burden. In addition, strengthening antemortem laboratory testing would lead to a confirmatory diagnosis in suspected encephalitic cases.

Over the period of 20 years, only 1155 dog bite victims received medical care across the 19 HF in the FCT with follow-up information, out of which 4.2% (n = 49/1,155) died of rabies. This finding highlights significant underreporting, consistent with a community survey in Kwara state that observed only 27% of dog bite victims sought care after a dog bite [36]. Elsewhere, previous rabies investigations indicated a huge disconnect between estimates from models and actual reported mortality data [2,6,37]. Reasons for this low reporting included victims often dying at home [6], misdiagnosis with cerebral malaria or other encephalitis [38], the utilisation of unorthodox methods of treatment [11], difficulty in obtaining samples from human victims of rabies and lack of capacity for routine testing [39]. Investment in enhanced surveillance applying a One Health strategy is crucial to overcoming this challenge. Moreover, community engagement to increase reporting is crucial. For example, a previous study in Nigeria has shown the contribution of community engagement in disease prevention and control at the community level [40]. Integrating traditional leaders in Acute Flaccid Paralysis (AFP) surveillance for polio yielded an excellent outcome for eliminating wild polio in Nigeria [40]. Incorporating such traditional healers, who are primary contacts after a bite in a rural setting might be valuable [41,42]. In addition, strengthening antemortem laboratory testing would lead to a confirmatory diagnosis in suspected encephalitic cases before being lost to follow-up. There is also the need to increase awareness at a local level using existing community structures in close collaboration with relevant authorities, including religious leaders, to ensure community buy-in and trust.

Some patients received PEP despite showing clinical signs of rabies. Considering its lack of therapeutic benefit, PEP is not recommended for individuals already showing rabies. Moreover, this intervention represents a waste of an expensive biomedical commodity, a threat of further RABV exposure to healthcare workers, and false hopes for the victim’s family.

Our results indicate an increased predicted probability of human rabies following a dog bite from free-roaming dogs compared to owned dogs. Previous studies indicate that free-roaming dogs are significant drivers of RABV perpetuation in endemic countries [43,44].

Free-roaming dogs are owned by individuals or the community but allowed to roam without supervision. Free roaming dogs often depend on humans for food and sometimes shelter and reproduction [45]. On the other hand, feral or stray dogs are ownerless dogs that no longer depend on humans [45]. Evidence suggests feral dogs account for a small proportion of free-roaming dogs in Africa [20].

The high turnover rates due to indiscriminate mating and human activities (e.g., open waste disposal) favour the survival of feral dogs. This is complicated further via the high chances of mixing with free-roaming dogs [15,46]. Clearly, this finding further heightens the challenge of free-roaming dogs to successful rabies prevention and control.

Innovative strategies to manage free-roaming dogs are needed. Oral rabies vaccination (ORV) has been successfully used to control wildlife rabies regionally, such as in Europe, the Americas, Israel, and South Korea. In addition, ORV has recently been piloted in Namibia [47,48]. Implementing ORV and complementing such an approach with capture and shelter for free-roaming dogs should support management efforts in an African context.

Moreover, alterations of certain local human activities, such as managing open garbage dumps by improved waste management systems, are needed. In collaboration with law enforcement officers in tandem with the existing local legal framework (leash laws) and responsible dog ownership via education, such actions would positively impact the community—decreasing dog bite occurrence and preventing human deaths. Creating vaccinated shelter dogs for adoption may create a sustainable system of managing a surplus of free-roaming dogs.

Conflict resolution that often ensues following a dog bite by an owned dog might be attributed to the predicted decrease in the probability of human rabies deaths. Such conflict resolutions often involve law enforcement officers (i.e., police) and risk assessment by professionals (i.e., veterinarians and physicians). This facilitates risk assessment and PEP administration for bitten individuals. Dog owners often bear the total cost of PEP if the offending dog is unvaccinated, and the bite is deemed unprovoked. This highlights the importance of implementing a legal framework and risk assessment in rabies prevention and control. Moreover, owned dogs are more likely to be vaccinated. Indeed, our results indicate an estimated decrease in the probability of human rabies deaths when victims were bitten by vaccinated dogs, highlighting the importance of canine vaccination in breaking the RABV transmission cycle toward preventing human deaths.

The odds of the risk of a human rabies death after individuals received PEP were predicted to decrease compared to no PEP. This finding agrees with a previous report from Tanzania, where the odds of a human rabies death following a canine RABV exposure were higher among individuals who did not undergo PEP [49], consistent with the known high efficacy of PEP. Four recent dog bite victims in Nigeria and Ghana died of rabies after failing to receive PEP [11,50,51]. These cases highlight a distinct minority of the over 20,000 people who die from rabies in Africa due to the cost of seeking care, poor availability of PEP, lack of understanding of the need for PEP, and low dog vaccination rates. Any potential canine RABV exposures in an endemic setting should be followed by a full-scale One Health risk assessment and immediate administration of wound care, rabies immune globulin (RIG) infiltration and vaccination, pending the outcome of the investigation. Moreover, PEP should be free and available in all H.F. Enhanced public education on the proper reporting channels and providing a dedicated mobile number for people to call in canine RABV exposures, with information on immediate first aid treatment of washing the bite wound with soap and water before presentation to a clinic, is necessary to help achieve the goals of the ZBT over the next several years.

A significant number of dog bite victims who received PEP had more than three doses of rabies vaccine (i.e., >75%). When considering updated WHO recommendations [52] and the evidence that virus neutralising antibodies are elicited by days 7–14 following prime-boost vaccination on days 0 and 7, such extra doses (i.e., >3) may not be as crucial if biologics are limited in supply, particularly if combined with timely medical care and infiltration of bite wounds with RIG. Such a finding underscores the need for education of physicians on the current WHO recommendation to curtail waste and maximise availability with at-risk communities, such as with the use of dose-sparing regimens and the value of intradermal administration.

The weak residual spatial dependency observed indicates that the coefficient standards errors and effect sizes are unlikely biased by non-independence-an evidence of the adequacy of our model in fitting the observed data. Our study has a few limitations that must be considered when interpreting our findings. Firstly, data on human rabies cases were based on clinical signs. Confirmatory diagnosis is unlikely due to cultural and social reasons and limitations for routine rabies testing, which is only available at the National Veterinary Research Institute [5]. We might have missed individuals who used traditional treatments, visited a pharmacy for self-care, and those who might have been buried immediately without reporting. We might have missed victims with long incubation periods. In addition, the time from exposure to seeking care (i.e., PEP) was unavailable as a risk factor. We only used the mean distance to H.F. as a proxy. We did not measure the impact of RIG administration, which should be infiltrated into and around wounds, according to WHO recommendations [52] because RIG is prohibitively expensive and has low availability throughout Nigeria (and much of Africa). We also lack data on the fate of the offending dogs to help in the risk assessment. Some of the landscape variables were unavailable for the 20 years covered by the study. Overall distances to hospitals might have changed over the course of the study. For the analysis, we only distinguished between owned and free-roaming because it is almost impossible to define if a dog is feral or free-roaming. Overall evidence suggests that only a small proportion of African free-roaming dogs are feral [45]. Recognition of these limitations allows refinement for future studies throughout the region to meet the goals of the ZBT. Our analysis demonstrated the practical application of a Bayesian approach to model sparse dog bites surveillance data with broad applications to other endemic rabies settings.

The low reporting noted in this study underscores the need for improved rabies surveillance in Nigeria to understand the burden for effective prevention and control. Incorporating traditional healers, who are primary contacts after a bite in a rural setting, might be valuable. In addition, strengthening antemortem laboratory testing would lead to a confirmatory diagnosis in suspected encephalitic cases before being lost to follow-up.

Our analysis has shown how to approach missing rabies data objectively and transparently, which is valuable for gaining inference using real-world data. Our modelling approach will be helpful for future applied research in Nigeria and low and medium-income countries with similar challenges. A further community-based investigation is pertinent to understand the risk among community members who might not report to the hospital after a dog bite and ensuing development of rabies.

Our findings highlight the importance of prompt and appropriate human PEP, canine vaccination, and responsible dog ownership in preventing human rabies deaths. Our findings underscore the need for innovative strategies to manage free-roaming dogs and advocate for responsible dog ownership.

## Supporting information

S1 File Trace plots showing the fits of the models to our observed, data most coefficients were drawn towards zero in model 1 (though mixing of chains was poor for some parameters as opposed to mode 2 with good mixing of chain. (DOCX)
