# Supplementary figures and images for: Modelling modifiable factors associated with the probability of human rabies deaths among self-reported victims of dog bites in Abuja, Nigeria
Source: PLoS Negl Trop Dis. 2023 Feb 21;17(2):e0011147. doi: 10.1371/journal.pntd.0011147 (PMC9983858; doi:10.1371/journal.pntd.0011147)

**Regularised horseshoe prior**


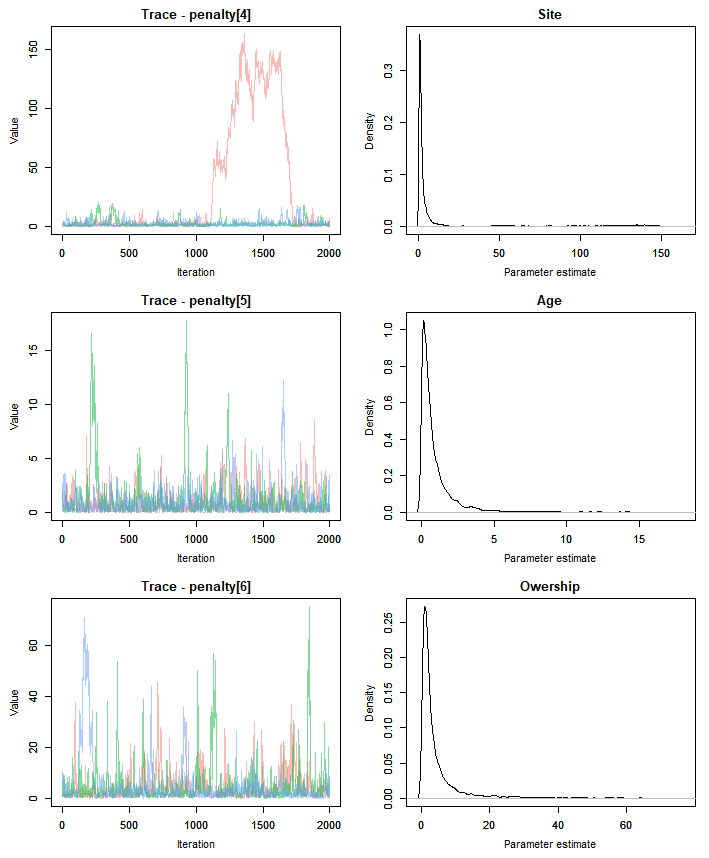


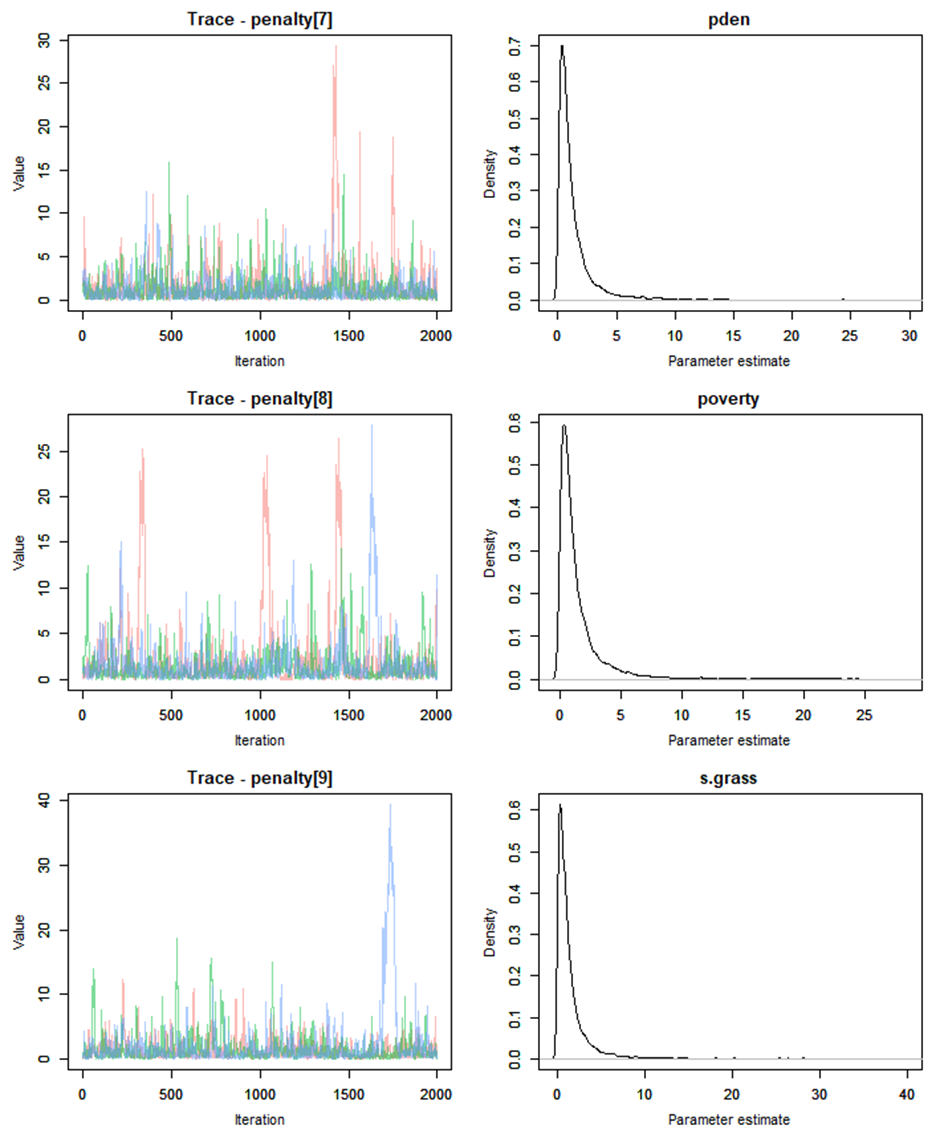


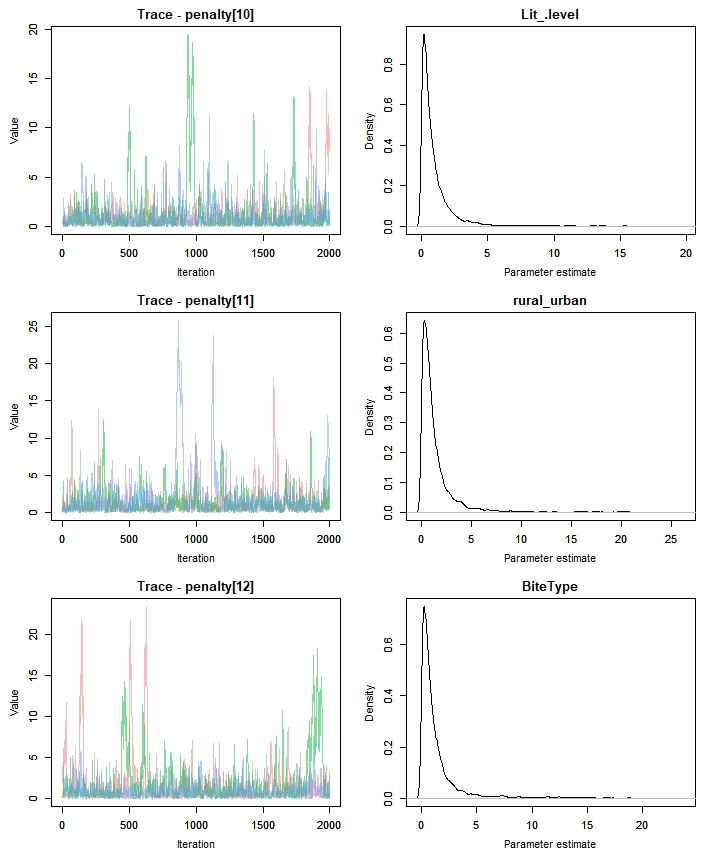


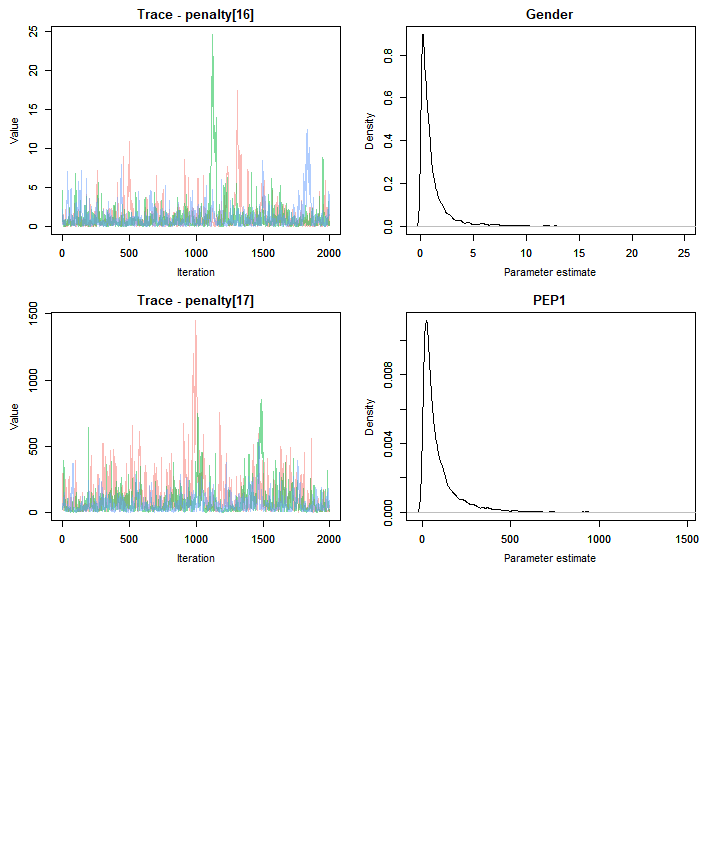


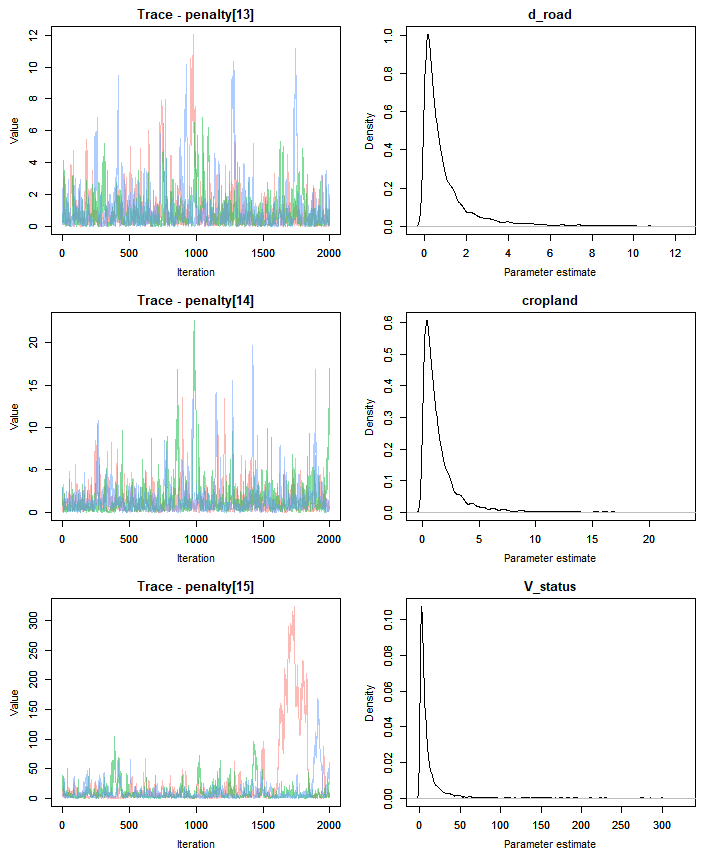


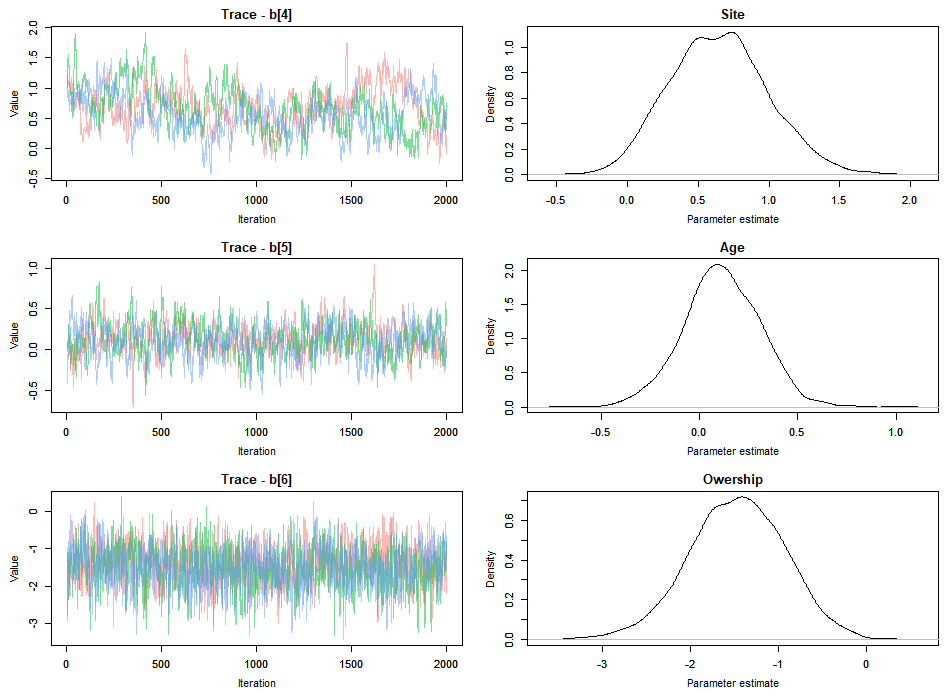

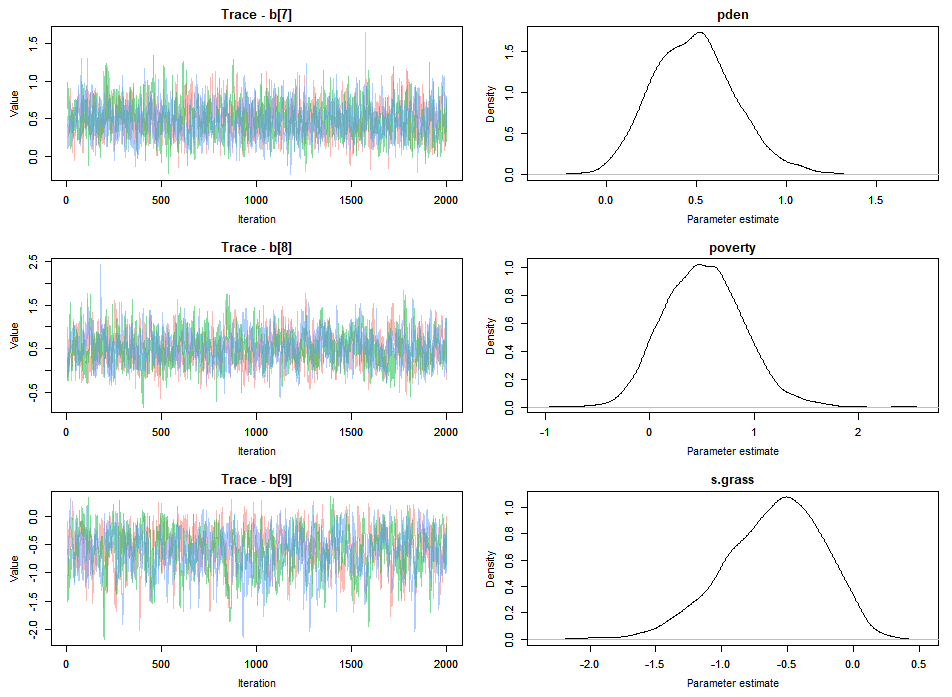

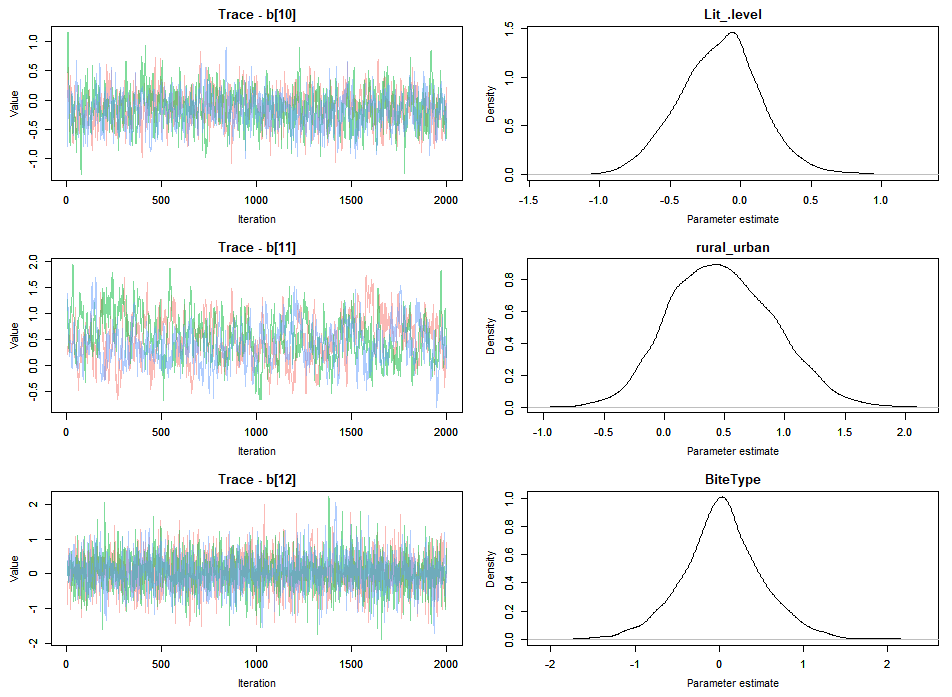

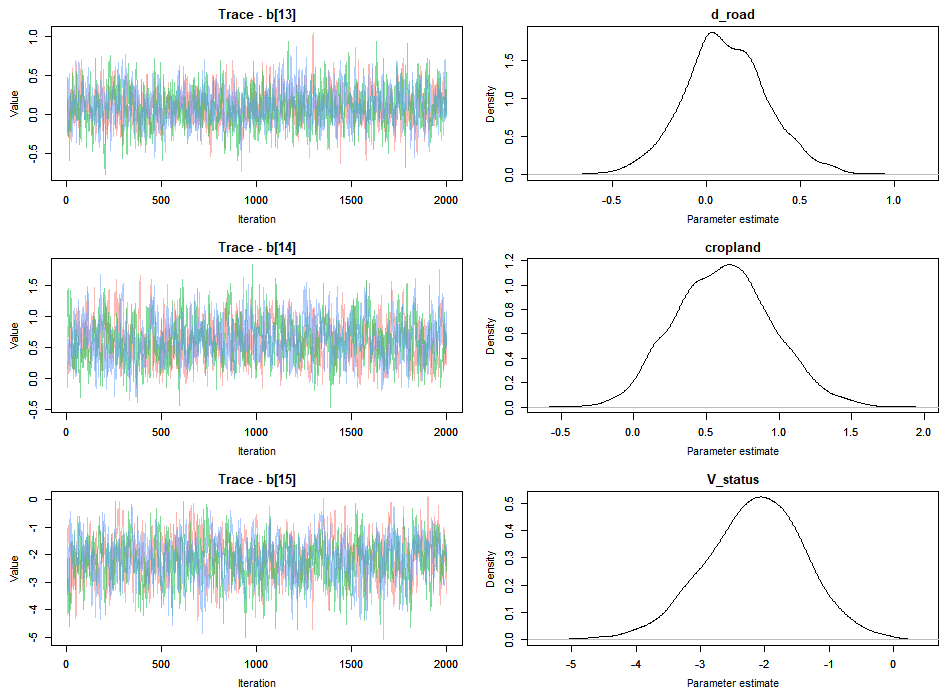

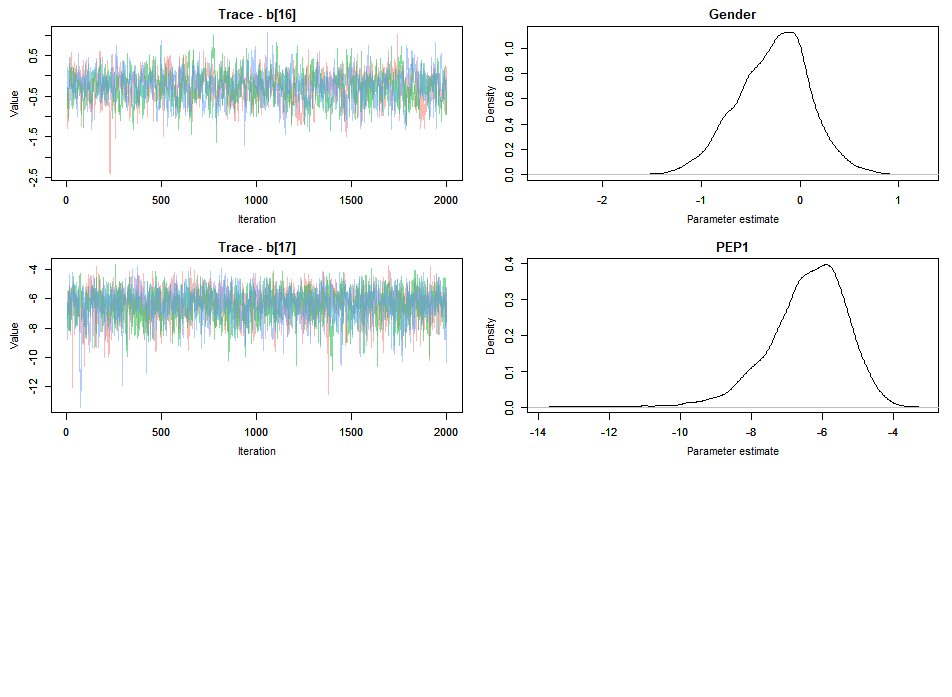

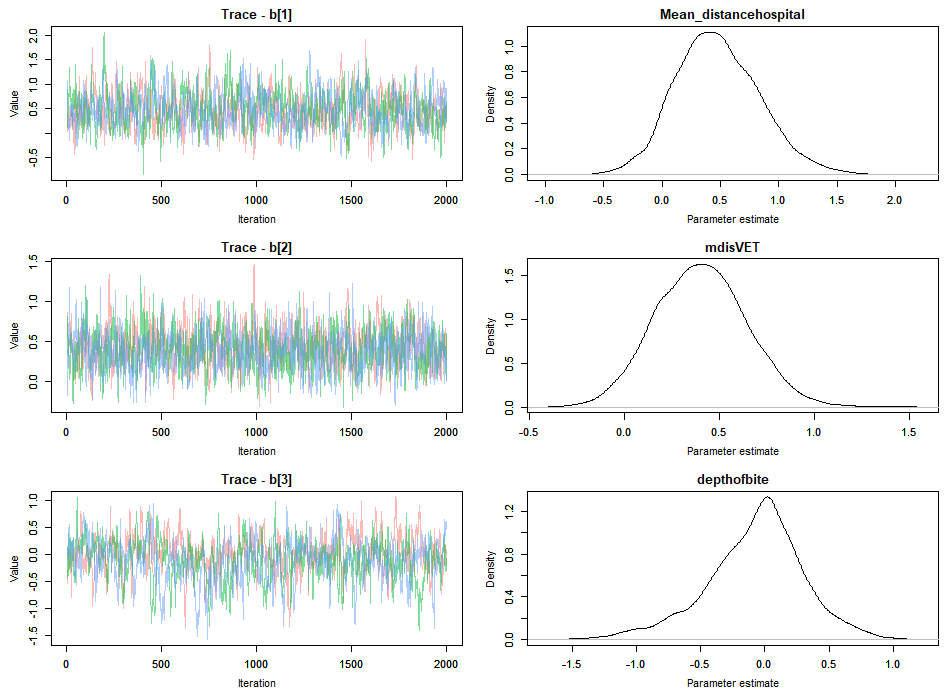

Supplement: S1 File — (DOCX) [file pntd.0011147.s001.docx]
